# Adverse reactions to acetaminophen and ibuprofen in pediatric patients: a narrative review

**DOI:** 10.1186/s13052-025-02135-z

**Published:** 2025-10-28

**Authors:** Francesca Vassallo, Massimo Martinelli, Linda Varcamonti, Pietro Buono

**Affiliations:** 1 Department of Translational Medical Sciences, Section of Pediatrics, University of Naples “Federico II”, Via Sergio Pansini, 5, Napoli, 80131 Italy; 2 Maternal and Child Care- Management Center, Napoli, 80143 Italy

**Keywords:** Acetaminophen, Ibuprofen, NSAIDs, Adverse reaction, Pediatrics

## Abstract

Acetaminophen and ibuprofen are among the most commonly used over-the-counter (OTC) medications for managing fever and pain in children. Although their safety profiles are well established, there has been a progressive increase in reports of suspected adverse drug reactions (ADRs) in pediatric populations in recent years. This trend may be partly attributable to improved reporting systems, but also to increased consumption. For example, the proportion of pediatric ibuprofen packages purchased without a prescription rose from 28% in 2008 to 70% in 2015. From 2020 to 2024, pediatric ibuprofen use grew by over 60%, and accordingly, the number of reported ADRs also increased. This rise may be due to specific pharmacovigilance programs targeting pediatric populations and the fact that, since 2009, ibuprofen in Italy no longer requires a prescription, making it more accessible and widely perceived as safe.

To provide a narrative review of suspected ADRs related to Acetaminophen and ibuprofen use in children.

A literature search was conducted using PubMed and Embase databases, employing the following terms: (Children OR Pediatrics) AND (Acetaminophen OR Ibuprofen OR NSAID OR Nonsteroidal Anti-Inflammatory Drugs) AND (Adverse Events OR ADRs).

A total of 337 records were identified, of which 15 studies were eligible for inclusion. According to Italian consumption data from the last five years, acetaminophen use declined from 68.8% in 2019 to 63.5% in 2024, while ibuprofen use increased from 31.2% to 36.7% (2). Additionally, the number of pediatric ibuprofen packages purchased increased by 61% between 2019 and 2024. Data from the European spontaneous reporting database (EudraVigilance) also showed a significant rise in reported ADRs in children receiving either drug. However, for comparable levels of use, ibuprofen appears to be associated with a higher rate of potentially serious adverse events.

Our analysis shows a marked increase in reported ADRs related to antipyretic use in children, likely linked to the rising use of ibuprofen in recent years. These findings emphasize the need for better parental education and healthcare provider guidance on the safe and appropriate use of antipyretics in pediatric patients.

## Background

Fever is one of the most common symptoms in children, accounting for approximately 20% of primary care and emergency department consultations [1]. Since fever often represent a physiological defense mechanism, all major guidelines recommend administering antipyretics only to alleviate the child’s discomfort [2].

Acetaminophen and ibuprofen are the most widely used over-the-counter medications for treating fever and pain in children [3]. While they offer comparable efficacy and safety at appropriate dosages—15 mg/kg for acetaminophen and 10 mg/kg for ibuprofen—they differ in their mechanisms of action and associated adverse effects. Acetaminophen is not a nonsteroidal anti-inflammatory drug (NSAID) but exhibits both antipyretic and analgesic properties. Ibuprofen, on the other hand, is a non-selective NSAID with antipyretic, analgesic, and anti-inflammatory activity.

Acetaminophen’s analgesic activity primarily involves central inhibition of prostaglandin synthesis via COX-2 and activation of descending serotonergic pain pathways. Because of its central mechanism, acetaminophen lacks many peripheral side effects typically associated with NSAIDs, such as gastrointestinal and renal toxicity. At therapeutic doses, it is generally well tolerated, though hepatotoxicity has been reported in cases of overdose, excessive or prolonged use, or concurrent administration of other acetaminophen-containing products [4].

In contrast, NSAIDs act mainly at peripheral sites of inflammation or tissue injury [5]. Ibuprofen inhibits both COX-1 and COX-2 enzymes, reducing pro-inflammatory prostaglandin synthesis. It is widely recommended alongside acetaminophen in pediatric populations due to its demonstrated safety and efficacy. However, both drugs can cause adverse effects. In the pediatric population, data on ADRs remain limited and scattered across studies. Reports of suspected ADRSs are publicly accessible through the European spontaneous reporting database maintained by EudraVigilance [6].

This review aims to assess the incidence of ADRs associated with acetaminophen and ibuprofen in children, in the context of increasing ibuprofen use in recent years.

## Methods

A comprehensive literature search was conducted using PubMed, MEDLINE, and Web of Science databases to identify relevant articles for this narrative review. Studies focusing exclusively on adult populations were excluded. The search strategy included the following keywords: (Child OR Pediatrics) AND (Acetaminophen OR Ibuprofen OR NSAID OR Nonsteroidal Anti-Inflammatory Drugs) AND (Adverse Events OR ADRs). In addition, reference lists of relevant review articles were manually screened to identify further studies.

## Results

**Fig. 1:**
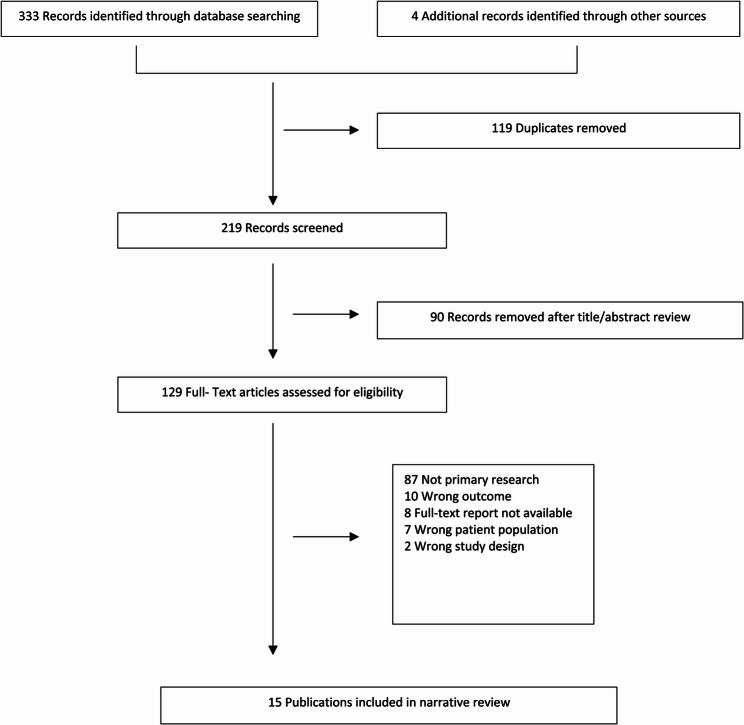
Flowchart of study Identification, Inclusion and Exclusion

**Fig. 2:**
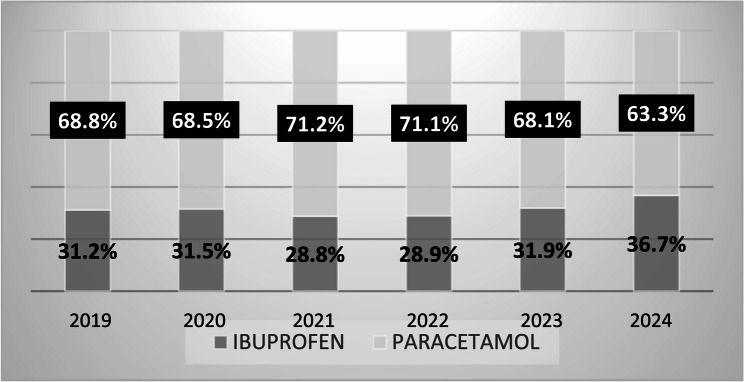
Acetaminophen and ibuprofen consumption in Italy in the last years. OsMed AIFA Data. Last access December 10, 2024

**Fig. 3:**
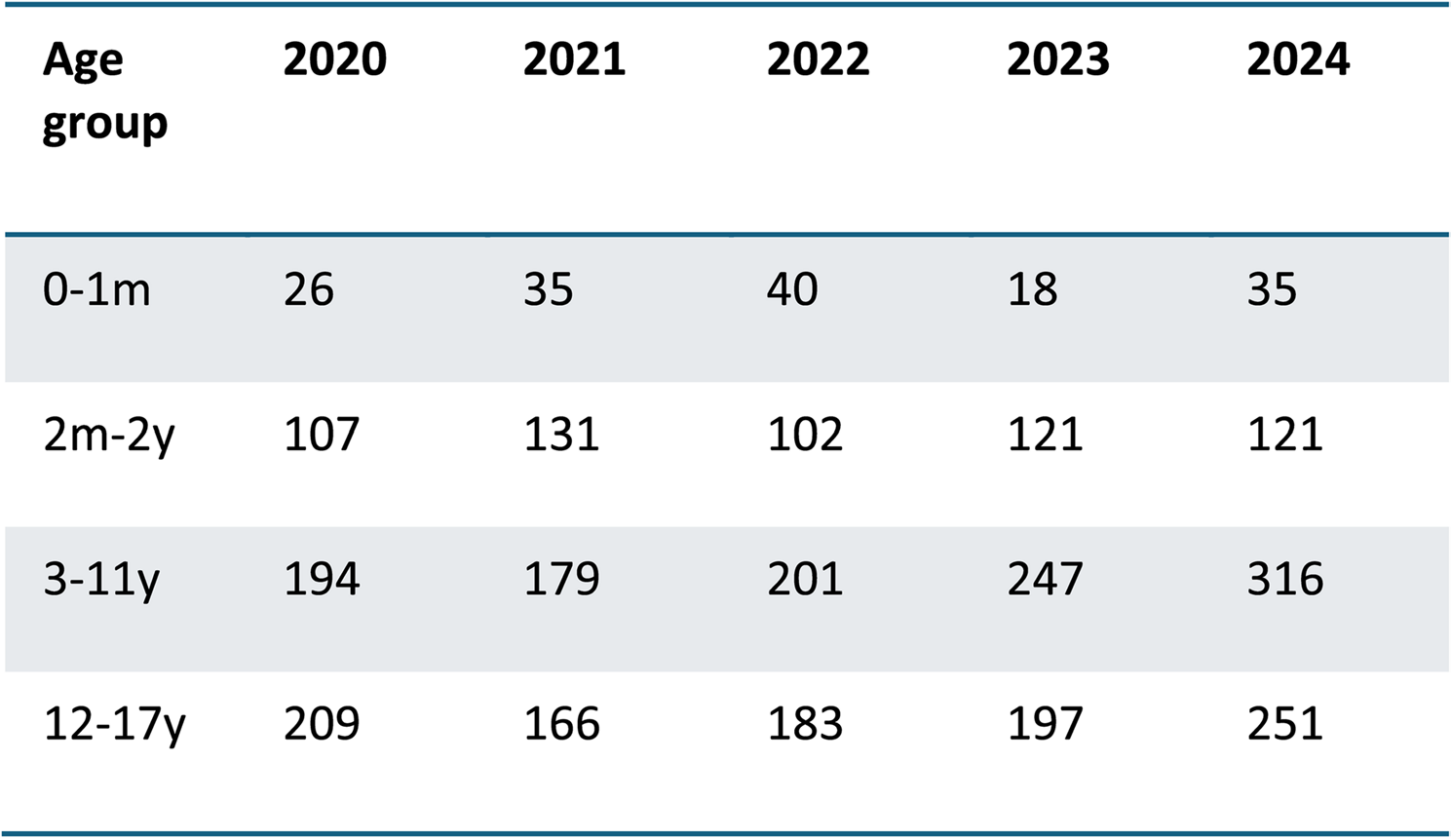
Number of Individual Cases of reported ADRs to ibuprofen by Age Group in the Last 5 Years. EudraVigilance data, Last access December 10, 2024

Of 219 records identified, 90 were excluded based on title and abstract screening, and 114 were excluded after full-text review. Consequently, 15 studies were included in the final analysis (Fig. 1). Adverse reactions to acetaminophen and ibuprofen in pediatric patients, as reported in the fifteen selected studies, were compared with drug consumption data over the past 5 years (Fig. 2). According to the European spontaneous reporting database (EudraVigilance), a total of 2,879 ADRs related to ibuprofen were reported between January 2020 and December 2024 (Fig. 3). While both acetaminophen and ibuprofen are generally safe and effective in children, it is crucial to adhere to the recommended dosages and administration intervals. Ibuprofen is also frequently used to treat inflammatory conditions, acute pain, and postoperative pain.

The most frequently reported adverse effects associated with acetaminophen and NSAIDs involve the gastrointestinal tract, including nausea, vomiting, abdominal pain, diarrhea, constipation, dyspepsia, and flatulence (Table 1). These symptoms are usually mild and self-limiting [3]. However, NSAIDs—more than acetaminophen—can occasionally lead to severe complications such as peptic ulcers, gastrointestinal bleeding, or perforation. A case-control study of 486 children aged 15 to 71 months examined the risk of upper gastrointestinal complications, including hematemesis and melena, linked to NSAID therapy, oral corticosteroids, and antibiotics [9]. Among hospitalized children, 66.6% were admitted for hematemesis, 6.4% for melena, and 6.0% for both symptoms. The remaining 21.0% showed signs suggestive of gastroduodenal lesions and were diagnosed with UGIC. Ibuprofen has also been associated with renal and hepatic adverse effects, particularly in cases of overdose, concomitant drug use, or pre-existing comorbidities [22].

**Table 1:** A summary of studies comparing adverse reactions to acetaminophen and ibuprofen in children

Reference	Type and Aim of the study	Methods	Keys results	Adverse events
Southey ER, 2009 [7]	Systematic review and meta- analysis of the clinical safety and tolerability of ibuprofen compared with acetaminophen in pediatric pain and fever	MEDLINE, Embase, Cochrane library	24 RCT examinated either ibuprofen and acetaminophen versus placebo for AE data. No significant difference between two group	Hepatic injury, gastrointestinal symptoms
AIFA Pediatric working group. 2010 [8]	Recommendations by the pediatric working group of AIFA on caution to use NSAID in children		About ibuprofen, the reports the reporting rate of RA reached 1.7 × 100,000 pediatric packages in 2010.	Acute renal failure (interstitial nephritis), rash, enterorrhages, GI bleeding
Bianciotto M, 2012 [9]	A case-control study on drug use and upper gastrointestinal complications in children	This study is part of a large Italian prospective multicentre study. The study population included children hospitalised for acute conditions through the emergency departments of eight clinical centres. Patients admitted for UGIC	Paracetamol showed a lower risk (adjusted OR 2.0, 95% CI 1.5 to 2.6) compared to ibuprofen (adjusted OR 3.7, 95% CI 2.3 to 5.9	NSAIDs, oral steroids and antibiotics, even when administered for a short period, were associated with an increased risk of UGIC.
Lee WJ, 2014 [10]	Observational study to describe the suspected medications and their AEs in children	All cases FDA adverse event reporting system (FEARS)	AEs report of the top 20 drugs: ibuprofen 72,3%, acetaminophen 68.8%	Gastrointestinal symptoms
Playne R, 2018 [11]	A randomized, single-blind, parallel group trial to evaluate safety of a paracetamol/ibuprofen fixed-dose combination in children	multicenter, randomized, single-blind, parallel group trial, 251 children aged 2–12 years undergoing day‐stay (adeno)tonsillectomy were randomized to two dose groups of the fixed-dose combination	The combination was well tolerated by both groups.	The most common adverse events were vomiting and nausea.
Tan E, 2020 [12]	Systematic review and meta- analysis to compare acetaminophen and ibuprofen for treatment of fever and pain in children younger than 2 years	All published studies from any health care setting or country that compared use of acetaminophen and ibuprofen in children with pain or fever	Very low rates of adverse events reported across all studies (kidney impairment, hepatotoxicity, asthma)	Uncomon. Acetaminophen and ibuprofen have similar serious adverse event profiles. Equivalent safety
Martinelli M. 2021 [13]	A national survey among Italian pediatricians about indications and adverse events of ibuprofen in children	Specific questionnaire-form regarding the management of ibuprofen therapy in children was distributed among a sample of pediatricians all over the Italian territory	Sixty-three (35%) out of 181 participating pediatricians reported 191 adverse events during ibuprofen administration.	GI bleeding being reported in 15.7%, epigastric pain in 15.1%, non-specified abdominal pain in 11.1% and nausea/vomiting in 11%. Severe adverse events including kidney damage (3.1%), complicated infections (0.5%), pneumonia associated empyema (0.5%), soft tissue infection (0.5%) and disseminated intravascular coagulation (0.5%)
Quaglietta L,2021 [14]	A narrative review in the era of COVID-19 pandemic on serious infectious events and ibuprofen administration in pediatrics	A literature search was performed including Medline- PubMed database	Ibuprofen should not be recommended for chickenpox management. Due to possible higher risks of complicated pneumonia, we suggest caution on its use in children with respiratory symptoms	Acute renal failure (interstitial nephritis), rash, enterorrhages, GI bleeding
Paul IM, 2021 [15]	Narrative review on acetaminophen and ibuprofen in the treatment of pediatric fever	Pubmed and Embase literature database to identify relevant articles.	Antypiretic effects of ibuprofen and acetaminophen are similar	No significant differences in rates of AEs between both group
Pelliccia V, 2022 [16]	Observational study to evaluate the ADRs of acetaminophen and ibuprofen over 15 years	Reporting database by AIFA (Pharmacolovigilance of the italian Drug Agency)	Acetaminophen ADRs in children were 15% of cases. Ibuprofen Pediatric ADRs were 26%	Skin and soft tissue in 63% of cases, gastrointestinal tract 47,5%, liver and kidney injury (6,7% and 2.3%)
Ziesenitz V.C. 2022 [17]	A comprehensive review of the literature of the past 20 Years on efficacy and safety of NSAIDs in infants	Summarizes the current knowledge on the safety and efficacy of various NSAIDs used in infants for which data are available,	Adverse drug reactions may be renal, gastrointestinal, hematological, or immunologic. even in young infants.	
Marano M. 2023 [18]	Retrospective study to analyze all the patients who contacted pediatric poison control center (PPCC) OPBG in Rome after exposure to acetaminophen and ibuprofen	Retrospectively reported the clinical data of children with accidental or intentional intake of inappropriate doses of acetaminophen/ibuprofen	Adverse event in 10% of cases with similar incidence in both group. A higher frequency of moderate intoxication in patient who took acetaminophen.	Neausea end vomiting most commonly reported.
Leitzen S,2023 [19]	Study to Compare Reports Collected in a Pharmacovigilance Project Versus Spontaneously Collected ADR Reports about ADR drug in children	systematically collected ADRs in the KiDSafe project and the spontaneous reports from EudraVigilance,	Reports from both data sources contributed to the identification of ADRs and dedicated issues related to drug therapy.	
Parri N. 2023 [20]	To evaluate the ADR to combination of paracetamol and ibuprofen in children		Safety profile is good	Nausea, vomiting, GI bleeding 4,4%
Castagno E, 2024 [21]	Paracetamol and ibuprofen combination for the management of acute mild‑to‑moderate pain in children: expert consensus using the Nominal Group Technique (NGT)	An investigation using the Nominal Group Technique was carried out between May and August 2022	The board achieved a final consensus on a better analgesic power of paracetamol and ibuprofen in fixed- dose combination as compared to monotherapy, without compromising safety.	no

## Discussion

Acetaminophen is a widely used analgesic and antipyretic with a well-established safety profile [23]. However, recent studies have reported a high frequency of accidental exposures in young pediatric patients, primarily due to unsupervised intake and medication errors [24].

When acetaminophen is administered at incorrect dosages, hepatic glutathione levels may become insufficient to detoxify N-acetil-p-benzochinoneimina (NAPQI), potentially leading to acute hepatic necrosis, renal tubular necrosis, and/or hypoglycemic coma [25]. Treatment typically involves the administration of N-acetylcysteine, which can prevent or attenuate hepatotoxicity.

According to our findings, the number of ADRs to ibuprofen reported in the Italian pharmacovigilance network AIFA (Agenzia Italiana del Farmaco) between March 2020 and December 2024 increased substantially [6].

NSAIDs have antipyretic, analgesic, and anti-inflammatory properties and are commonly prescribed in pediatric patients for a range of indications, including fever, postoperative pain, and inflammatory disorders such as juvenile idiopathic arthritis (JIA) and Kawasaki disease. Their primary mechanism of action is the inhibition of prostaglandin biosynthesis via the blockade of COX-1 and COX-2 enzymes [26]. Adverse events following ibuprofen use in children have been reported, including its possible role in exacerbating the clinical course of infectious diseases. Notably, ibuprofen has been linked to severe necrotizing soft tissue infections (NSTIs) during varicella, and has been associated with an increased risk of complicated pneumonia when used prior to hospitalization. While data exist for septic pediatric patients, ibuprofen has been considered safe and effective in managing cystic fibrosis (CF) flare-ups. Currently, there is no available regarding ibuprofen use during COVID-19.

A 2021 study by National Institute for Health and Care Excellence (NICE) confirmed that NSAIDs are highly effective for acute pain management, especially when used in combination with acetaminophen. Nevertheless, their contraindications and potential adverse effects should be carefully assessed prior to administration [27].

**Fig. 4:**
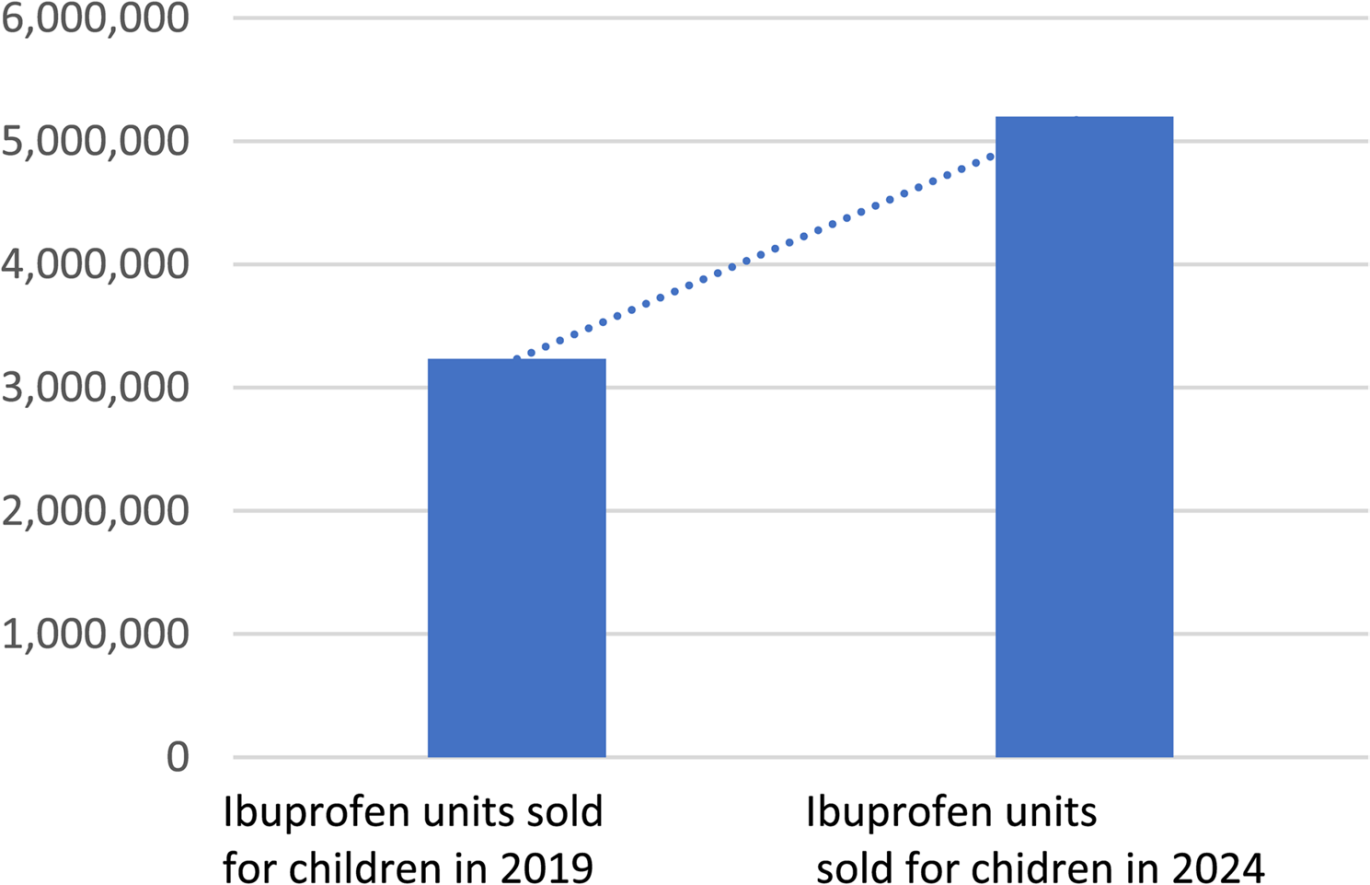
OsMed AIFA Data. Last access December 10, 2024 [32]

Since 2010, the AIFA Pediatric Working Group has observed a growing number of suspected ADRs related to ibuprofen, a trend that coincides with its increased over-the-counter availability [8]. Over the past decade, numerous studies have documented ibuprofen-related ADRs [28–31]. In Italy, consumption data over the last five years reveal a decline in acetaminophen use (from 68.8% in 2019 to 63.5% in 2024), while ibuprofen use has risen (from 31.2% in 2019 to 36.7% in 2024) [2] (Fig. 4). Furthermore, the number of pediatric ibuprofen packages sold in Italy has increased by 61% since 2019, contrasting with trends observed in other European countries [32].

In 2021 a survey investigating ibuprofen-related ADRs was conducted via questionnaires administered to family pediatricians. Of the 181 respondents, 63 (35%) reported a total of 191 ADRs associated with ibuprofen use [13]. Gastrointestinal issues were the most commonly reported, including gastrointestinal bleeding in 30 cases (15.7%), epigastric pain in 29 (15.1%), unspecified abdominal pain in 22 (11.1%), and nausea or vomiting in 21 cases (11%). In a 2016 multicenter retrospective study, Cardile et al. examined data from eight Italian pediatric gastroenterology centers (January 2005–January 2013) to characterize NSAID-related gastrointestinal bleeding [29]. The study identified 51 pediatric patients with GI bleeding, with ibuprofen being the most frequently implicated NSAID. The authors concluded that NSAID-induced GI bleeding is not uncommon and is frequently linked to misuse, including self- medication.

A 2023 study reported that acute kidney injury (AKI) associated with NSAID use can occur even at therapeutic doses, especially in dehydrated children. Follow-up revealed a significant risk of progression to chronic kidney disease. Therefore, NSAIDs administration is strongly discouraged in children experiencing dehydration [33].

The literature further suggests that NSAIDs may worsen bacterial and viral infections, particularly skin and soft tissue infections (SSTIs) [14]. A 2009 multicenter case-control study by Legras et al. examined whether NSAID use influenced the severity of community-acquired bacterial infections. Among adults, NSAID use was associated with an increased risk of severe sepsis or septic shock [34]. NSAIDs are known to inhibit oxidative chemotaxis, phagocytosis, and bacterial clearance, as well as to impair granulocyte degranulation and lymphocyte function.

A multicenter retrospective cohort study by Nicollas et al. found that ibuprofen may increase the risk of orbital and/or intracranial complications in children with acute frontoethmoidal sinusitis. Consequently, ibuprofen use should be avoided when acute sinusitis is suspected in pediatric patients [35]. In April 2019, the French National Agency for the Safety of Medicines and Health Products (ANSM) issued a warning against NSAID use in infectious diseases, following a 20-year analysis of real-world safety data on ibuprofen and ketoprofen [36]. Most complications were associated with streptococcal infections and developed within 2–3 days of NSAID starting. As a result, ANSM released practical guidelines recommending the use of NSAIDs at the lowest effective dose for the shortest possible duration. These recommendations have been endorsed by the AIFA Pediatric Working Group [8].

Marano et al. (2023) analyzed cases of pediatric patients presenting to the Poison Control Center at Bambino Gesù Children’s Hospital in Rome between January 1, 2018, and September 30, 2022, following unintentional, accidental, or intentional ingestion of inappropriate doses of acetaminophen and/or ibuprofen. Their findings demonstrated that even these commonly perceived “safe” drugs can lead to toxicity and adverse reactions if improperly administered [18].

ADRs and medication errors in children may result from a lack of age-appropriate formulations, incorrect dosing, or unsuitable pharmaceutical preparations. Moreover children may have different pharmacodynamic and pharmacokinetic responses compared to adults. Therefore, the reporting of pediatric ADRs is essential for expanding knowledge on drug safety in this population. However, variation in data collection methodologies complicate direct comparisons.

A study by Leitzen et al. compared ADRs reported in in the KiDSafe pharmacovigilance project with those spontaneously submitted to EudraVigilance. Ibuprofen was identified as the most frequently implicated drug in ADRs requiring hospitalization [19].

## Conclusions

Our analysis of the selected studies highlights a substantial increase in reported adverse events among children treated with antipyretics, likely reflecting the growing use of ibuprofen over the past decade. Although most ADRs were mild and frequently linked to dosing errors (e.g., incorrect dose, frequency, or treatment duration), these findings underscore the critical need for increased awareness among healthcare professionals and caregivers regarding the proper use of NSAIDs in pediatric patients—particularly in high-risk groups such as dehydrated children, preterm infants, those with low birth weight, or children receiving concomitant therapies. These observations emphasize the importance of parental education concerning the appropriate administration of antipyretics. Further well-designed prospective studies are warranted to validate these findings and provide robust data on the long-term safety profile of NSAIDs in pediatric population.

## Data Availability

Not applicable.

## Abbreviations

ADRs: Adverse Drug Reactions
NSAID: Non Steroidal Anti-Inflammatory Drug
COX-1 COX-2: Cyclooxygenase-1, Cyclooxygenase-2
UGIC: Upper Gastro-Intestinal Complication
AIFA: Agenza Italiana del Farmaco
JIA: Juvenile Idiopathic Arthritis
NSTIs: Necrotizing Soft Tissue Infections
NICE: National Institute for Health and Care Excellence
AKI: Acute Kidney Injury
SSTIs: Skin and Soft Tissue Infections
ANSM: French National Agency for the Safety of Medicines and Health Products
